# Aircraft maintenance and mesothelioma

**DOI:** 10.4103/0019-5278.64613

**Published:** 2010

**Authors:** Claudio Bianchi, Tommaso Bianchi

**Affiliations:** Center for the Study of Environmental Cancer–Italian League against Cancer, Monfalcone Hospital, Monfalcone - 34074, Italy

Dear Sir,

A variety of toxic substances have been used in aircraft construction.[1] Asbestos-containing materials were present in various aircraft components.[2] Asbestos content was relatively high in the brakes, ranging from 16 to 23% by weight in some types.[2] This suggests that people employed in aircraft maintenance-repair work, including brake replacement, might be at risk for asbestos disease. We reviewed a case of malignant pleural mesothelioma developed in a 63-year-old man. As an aeronautical engineer, he was involved in aircraft maintenance-overhaul at the airports of Naples and Rome in the period 1963-2003. He was treated by right pleuropneumonectomy for mesothelioma in October 2003, and died for mesothelioma recurrence 11 months later. Some studies indicate an increased mortality for pleural cancer among people employed in aircraft industry.[1,3] Regarding aircraft maintenance-repair, further mesothelioma cases have been observed in Italy in this branch, besides the present one. In 1996, a pleural mesothelioma was histologically diagnosed at the Rome University in a 62-year-old man who had worked as an aircraft mechanic at the Rome airport for 35 years. At the Mesothelioma Registry of Campania, two cases of pleural mesothelioma have recently been reported with histories of maintenance-repair work at the Naples airport.[4] In all the above cases, mesothelioma was defined as an occupational cancer, asbestos related.

A recent study, performed in Georgia, USA, on airborne asbestos exposure occurring during light aircraft brake replacement, did not show alarming levels of asbestos fibers.[2] However, it is probable that the situation observed in such study does not reflect the work practices occurred in the past, and even currently occurring, in a majority of countries. Given the large number of people employed in aircraft maintenance-repair work throughout the world, mesothelioma in such branch could represent a considerable problem.
